# Spontaneous regression of peritumoral brain edema in an asymptomatic meningioma: A radiological case report

**DOI:** 10.1016/j.radcr.2026.04.099

**Published:** 2026-06-06

**Authors:** Toshihide Takahashi, Kiyoyuki Yanaka, Hitoshi Aiyama, Saaya Maruyama, Nobuyuki Takahashi, Aiki Marushima, Eiichi Ishikawa

**Affiliations:** a Department of Neurosurgery, Tsukuba Memorial Hospital, Kaname 1187-299, Tsukuba, Ibaraki, Japan; b Department of Radiology, Tsukuba Memorial Hospital, Kaname 1187-299, Tsukuba, Ibaraki, Japan; c Department of Neurosurgery, Institute of Medicine, University of Tsukuba, Tennodai 1-1-1, Tsukuba, Ibaraki, Japan

**Keywords:** Active surveillance, Brain edema, Meningioma, Spontaneous regression

## Abstract

Peritumoral brain edema (PTBE) is a common radiologic finding in meningiomas and often influences clinical decision-making. However, spontaneous regression of PTBE without intervention is rare. We report a 74-year-old man with an incidentally detected right middle cranial fossa meningioma accompanied by marked PTBE following minor head trauma. The patient remained asymptomatic and was managed conservatively without steroids or surgery. Over a 3-year follow-up period, the tumor size remained stable, whereas the extensive PTBE regressed almost completely on serial MRI. This case suggests that even substantial PTBE may resolve spontaneously and highlights the importance of individualized management strategies in asymptomatic meningioma patients.

## Introduction

Meningiomas are considered the most common primary intracranial tumors and are typically benign and slow-growing [1]. Despite their generally indolent nature, their clinical manifestations are often influenced by peritumoral brain edema (PTBE), which can result in significant neurological symptoms and complicate surgical management [2,3,4]. PTBE in meningiomas represents vasogenic edema resulting from blood–brain barrier disruption and increased vascular permeability, which are frequently associated with tumor-secreted factors such as vascular endothelial growth factor (VEGF), interleukin-6 (IL-6), and matrix metalloproteinases (MMPs) [[4], [5], [6]]. The presence and severity of PTBE are commonly used as indirect markers of tumor aggressiveness and determinants for early surgical or corticosteroid intervention. However, the natural history of PTBE remains unclear. The spontaneous regression of edema without any treatment is exceptionally rare [[7], [8], [9]]. Previous studies have reported a spontaneous regression of the meningioma itself, but reports focusing specifically on the spontaneous resolution of a major secondary effect—namely, associated PTBE—and its underlying mechanisms have been limited [10].

This study reports an asymptomatic right middle cranial fossa meningioma that showed a significant spontaneous regression of PTBE over a 3-year observation period, despite the absence of medical or surgical intervention. Furthermore, this case provides insights into the dynamic and potentially reversible nature of PTBE and suggests important implications for conservative management strategies in appropriately selected meningioma patients.

## Case report

This study investigated a 74-year-old man who presented to our department a few days after sustaining a minor head injury. The patient underwent computed tomography (CT) scan as part of routine evaluation after minor head trauma, despite the absence of neurological symptoms. A CT scan performed as part of the acute evaluation revealed an incidental dural-based extra-axial mass in the right middle cranial fossa (Fig. 1A). A subsequent contrast-enhanced magnetic resonance imaging (MRI) confirmed a 3-cm, well-circumscribed, homogeneously enhancing lesion consistent with a meningioma (Figs.1B–D). The mass showed iso-intensity on T1-weighted imaging, faint high intensity on T2-weighted imaging, and strong homogeneous enhancement on contrast-enhanced T1-weighted imaging. Notably, a significant T2-hyperintense peritumoral edema involving the adjacent right temporal lobe was observed. Despite extensive edema, the patient showed no neurological symptoms, including seizures, aphasia, or cognitive decline, and the findings of his physical and neurocognitive examinations were entirely normal. Although venous outflow disturbance has been reported as an important contributor to PTBE, definitive evidence of tumor-related venous compression was not demonstrated in this case [11]. The observed focal narrowing was located away from the tumor, and therefore its contribution remains uncertain. Although focal narrowing was observed, it was not located adjacent to the tumor but rather in a more distal convexity segment.

**Fig. 1:**
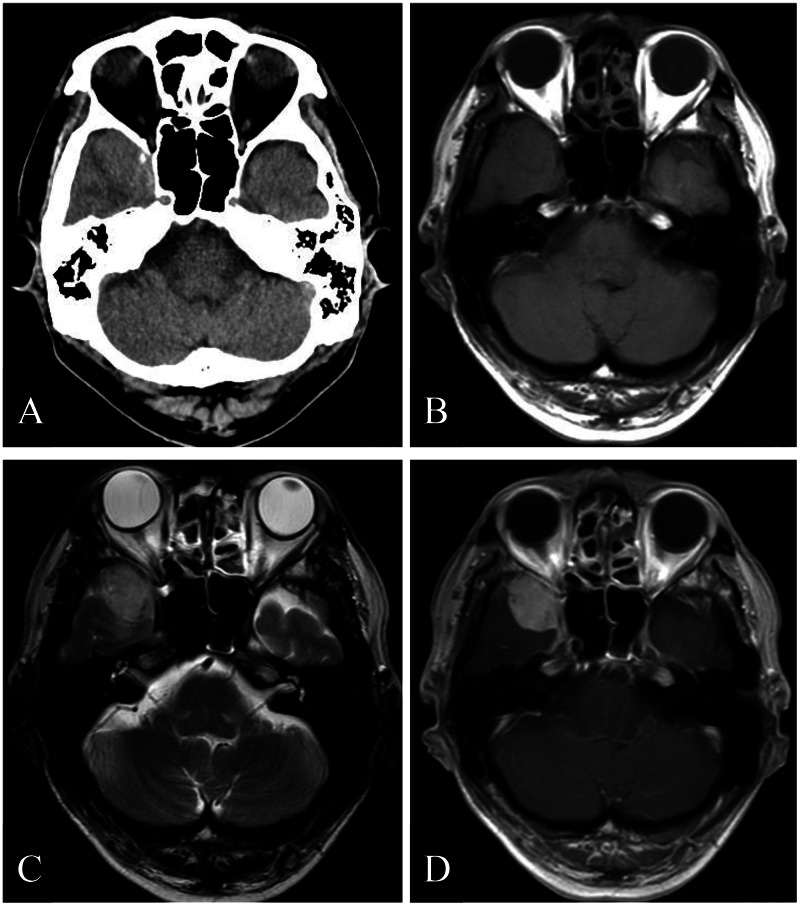
Initial diagnostic axial imaging at outpatient presentation. (A) Non-contrast CT showing an iso-dense extra-axial mass in the right middle cranial fossa. CT, computed tomography. (B) T1-weighted MRI indicating that the lesion was iso-intense relative to the cortex. MRI, magnetic resonance imaging. (C) T2-weighted MRI revealing faint hyperintensity within the mass and surrounding PTBE in the adjacent temporal lobe. PTBE, peritumoral edema. (D) Contrast-enhanced T1-weighted MRI indicating a strong, homogeneous enhancement, which is consistent with the diagnosis of meningioma.

**Fig. 2:**
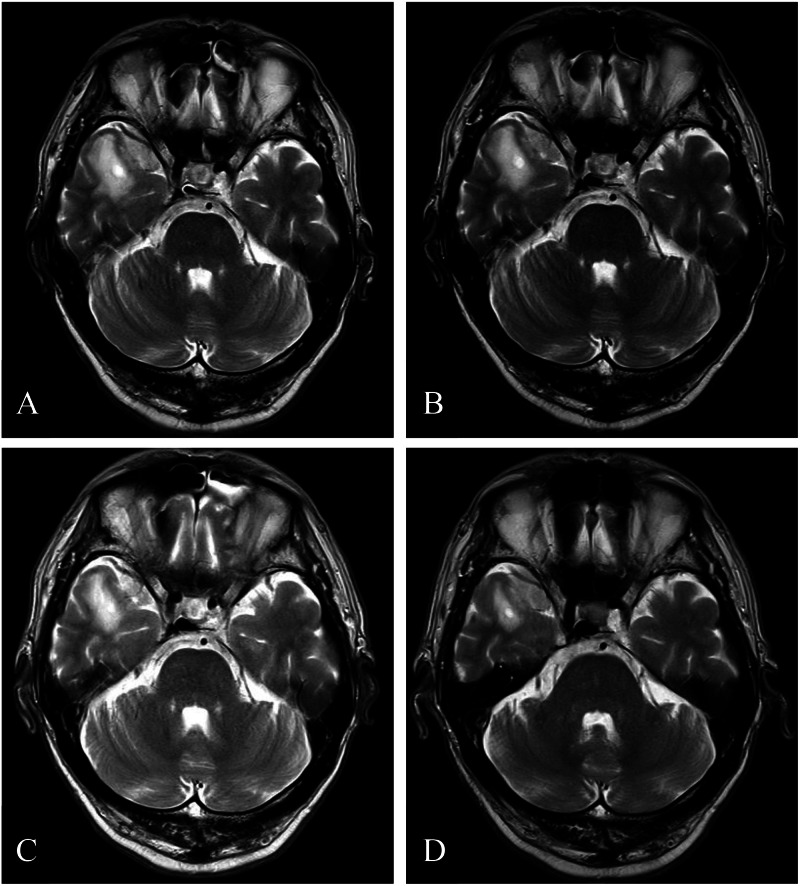
The serial axial T2-weighted magnetic resonance images showing the spontaneous regression of PTBE. PTBE, peritumoral brain edema. (A) At the time of initial presentation, a high-intensity area was observed in the right temporal lobe adjacent to the tumor. (B) Six months after the initial presentation, the extent of T2 hyperintensity indicates the early evidence of reduction. (C) At 1 year of follow-up, the edema had substantially decreased compared with the initial presentation, although the tumor size remained stable. (D) At 3 years of follow-up, the edema has largely regressed, leaving only a residual trace amount of T2 hyperintensity.

Since the tumor had been discovered incidentally during his head injury workup and the patient remained entirely asymptomatic, an active surveillance strategy was initiated. An MRI follow-up was performed every 6-12 months over the next 3 years. Importantly, the patient was not administered corticosteroids or any other anti-edema therapy during this period.

Throughout the 3-year observation period, the patient remained neurologically stable and asymptomatic. The serial MRI scans indicated minimal changes in tumor size but showed a gradual, significant reduction in peritumoral edema, which had markedly regressed by the third year, with only a trace residual hyperintensity remaining (Figs. 2A–D). A small cystic lesion with a punctate isointense focus was observed within the edematous region (Fig. 3A–D), the significance of which remains unclear but may represent a focal fluid collection or degenerative change. As the edema resolved, the cerebrospinal fluid (CSF) cleft sign (initially obscured) became more clearly visible around the tumor margin, although the definitive assessment of widening was limited by slice variability. Moreover, the lesion itself remained approximately 3 cm in diameter, with no new enhancement or evidence of hemorrhage or necrosis. Serial measurements confirmed that the tumor size remained stable throughout the follow-up period. This spontaneous resolution of PTBE, occurring without any therapeutic intervention during the 3-year follow-up period, was considered the defining radiologic feature of this case.

**Fig. 3:**
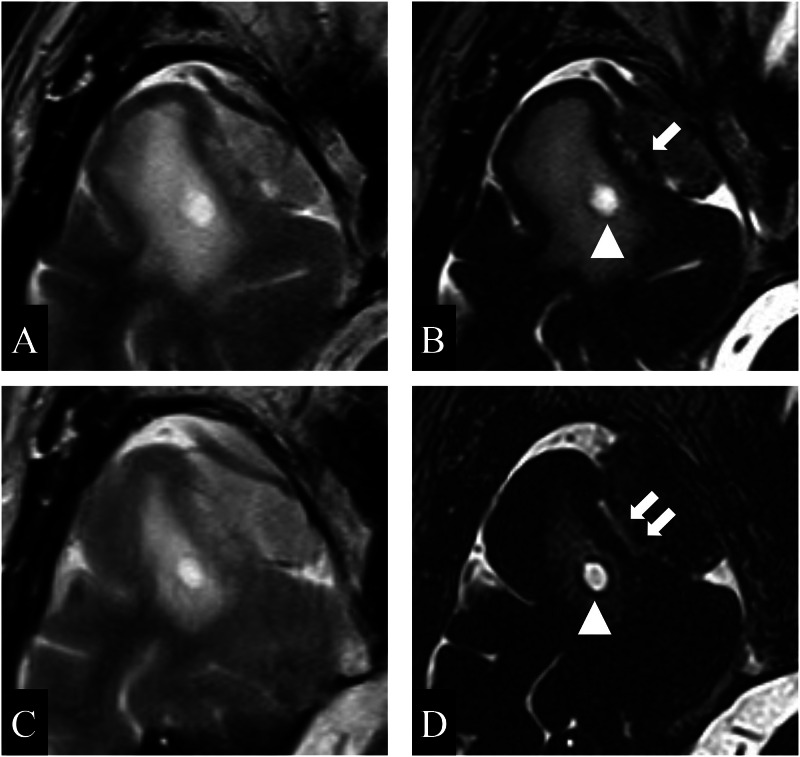
The magnified views of the tumor–brain interface and edema reduction. (A, C) The axial T2-weighted images at 6 months of follow-up (A) and 2 years of follow-up (C). Vasogenic edema at the right temporal tip (T2 hyperintensity) indicated a significant interval reduction between these time points. (B, D) Corresponding heavily T2-weighted images. In (B), the cerebrospinal fluid (CSF) cleft (white arrow) appears partially effaced and discontinuous. In (D), the CSF cleft (double arrow) appears more continuous. A small cystic lesion with a punctate isointense focus (arrow head) is observed within the edematous region adjacent to the tumor, the significance of which remains unclear.

## Discussion

Although the exact pathophysiological mechanisms underlying the spontaneous regression of PTBE remain unclear, several plausible explanations can be proposed. Among them, the impairment and subsequent restoration of venous drainage is considered one of the most important mechanisms. PTBE has been shown to develop as a consequence of venous congestion caused by the tumor-related compression of cortical veins or major draining pathways, including the superficial middle cerebral vein (SMCV). Previous studies have proven a strong association between SMCV compression and the severity of PTBE, particularly in meningiomas arising from the sphenoid wing or middle cranial fossa [11]. According to this concept, the gradual relief of venous outflow obstruction—either through subtle decompression or the development of collateral venous pathways—may improve regional perfusion and result in the progressive resolution of edema [3,12].

In the present case, although the SMCV drainage pattern was classified as a cavernous type, the segment showing apparent narrowing was found slightly away from the tumor itself, making the direct mechanical compression by the tumor unclear. Nevertheless, venous outflow disturbance remains a relevant and necessary component in explaining the significant PTBE observed at presentation, while the improvement in venous hemodynamics likely contributed, at least in part, to the subsequent edema regression.

Another possible mechanism involves the changes in the tumor’s biological activity. Meningiomas have been known to secrete VEGF and other cytokines that tend to increase vascular permeability. A reduction in the expression of these factors—whether it is due to alterations in tumor biology or cellular senescence over time—could restore vascular integrity and reduce ongoing vasogenic edema formation [13,14].

In addition to these factors, the changes at the tumor–brain interface may have played a pivotal role in this case. Although the definitive assessment of the CSF cleft widening was limited by slice-to-slice variability in serial MRI, the CSF cleft appeared subjectively clearer and more continuous over time. Rather than being a secondary epiphenomenon, the reconstruction of the CSF cleft may represent a key upstream event linking multiple pathogenic pathways. The re-establishment of the arachnoid and pial layers at the tumor–brain interface could serve as a structural barrier, compromising the diffusion of the tumor-secreted permeability factors into the adjacent brain parenchyma and facilitating the restoration of the blood–brain barrier function [15]. Moreover, the reformation of the CSF cleft may also reduce the mechanical stress exerted by the tumor on the adjacent cortical and bridging veins, including the SMCV and its tributaries. In this context, the improvement of venous drainage may be viewed not as an isolated mechanism but rather as a downstream consequence of the restored separation between the tumor and brain. Thus, a CSF cleft reconstitution may provide a more comprehensive explanation for the spontaneous regression of PTBE in this case, integrating both vascular permeability and the venous outflow components. Although contrecoup injury could theoretically contribute to temporal lobe changes, the long-term persistence and gradual regression of edema in this case are not consistent with typical traumatic edema patterns.

Additional factors, such as vascular remodeling or adaptive improvement in cerebrovascular autoregulation associated with aging, may normalize the microcirculatory dynamics and decrease the susceptibility to vasogenic edema [16]. Although these mechanisms remain unclear, they still support the concept that PTBE is a dynamic and multifactorial process rather than a fixed, purely mechanical phenomenon.

From a clinical perspective, this study proposes several important implications. PTBE is a significant radiologic and clinical finding, which is often associated with symptoms of increased intracranial pressure and a higher incidence of seizures [4]. Nonetheless, the findings of this study suggest that not all instances of PTBE require intervention; even substantial edema may remain clinically silent and resolve spontaneously. This supports the safety of conservative management in carefully selected asymptomatic patients with stable meningiomas, for whom close MRI surveillance represents a reasonable alternative to surgery or corticosteroid therapy, thereby avoiding unnecessary iatrogenic risks.

Moreover, the dissociation between a stable tumor size and progressive edema regression suggests that PTBE reflects not only a mechanical mass effect but also dynamic tumor–vascular interactions that may self-correct over time. Thus, recognizing the potential for spontaneous PTBE regression is important for radiologists and clinicians, preventing the misinterpretation of persistent or fluctuating edema as an indicator of tumor progression or malignant transformation.

A key limitation of this report is the absence of histological confirmation, as the patient was managed conservatively without surgery or biopsy. However, the characteristic radiologic features strongly confirm the diagnosis of meningioma. Imaging-based diagnosis has been widely accepted in typical cases when surgical intervention is not indicated, and the long-term radiologic stability observed in the present case further reinforces this interpretation. This case emphasizes that PTBE should not be interpreted solely as an indicator of tumor aggressiveness and may not always warrant immediate intervention.

## Conclusion

This study shows the spontaneous and significant regression of PTBE in an untreated, asymptomatic meningioma over a 3-year period. These findings emphasize that PTBE is a dynamic and potentially reversible process that does not necessarily require immediate intervention. Furthermore, the acknowledgment of this phenomenon may support the individualized, conservative management strategies for appropriately selected asymptomatic patients, provided that they are closely monitored with serial imaging.

## Patient consent

Informed consent was obtained from the patient for publication of this case report and accompanying images.
